# A Potential Probiotic *Lactiplantibacillus Plantarum* Isolate from Egyptian Cottage Cheese Alleviates Metabolic Syndrome Manifestations: In Vitro and In Vivo Characterization

**DOI:** 10.1007/s12602-025-10896-6

**Published:** 2026-01-24

**Authors:** Yehya Abdel-Moniem, Kareem A. Ibrahim, Omneya M. Helmy, Mona T. Kashef

**Affiliations:** 1 Post Graduate Program, Faculty of Pharmacy, Cairo University, Cairo, 11562 Egypt; 2 Department of Microbiology and Immunology, Faculty of Pharmacy, Egyptian Russian University, Suez Road, Cairo, 11829 Egypt; 3 Department of Microbiology and Immunology, Faculty of Pharmacy, Cairo University, Cairo, 11562 Egypt

**Keywords:** Hypocholesterolemic effect, Hypoglycemic effect, Lactic acid bacteria, *Lactiplantibacillus plantarum*, Metabolic syndrome, Probiotics

## Abstract

Isolation of beneficial probiotics from traditional foods is a priority in functional food research. We isolated and characterized a lactic acid bacteria (LAB) probiotic strain from Egyptian cuisine, with therapeutic applications for alleviating the manifestations of metabolic syndrome (MetS), including hyperglycemia, hypercholesterolemia and obesity. LAB (*n* = 10) were isolated from 12 food and juice samples, identified and assessed in vitro for glucose- and cholesterol-lowering capabilities. The most promising isolate underwent probiotic characterization, including gastrointestinal tolerance, surface hydrophobicity, auto-aggregation, and milk fermentation capacity. Other beneficial properties, such as exopolysaccharide production and antimicrobial activity, were also tested. The selected isolate was evaluated for a hypoglycemic and hypocholesterolemic effect using a high-fat diet/streptozotocin-induced hypercholesterolemia and diabetes model in Wistar rats. *Lactiplantibacillus plantarum* Y_10b_, isolated from Egyptian cottage cheese, reduced glucose and cholesterol levels in vitro by 53 ± 0.47% and 98 ± 0.18%. It showed good probiotic characteristics: minimal viability loss in simulated gastrointestinal conditions (0.07 and 0.08 log_10_ CFU/mL), good hydrophobicity (> 70%), high auto-aggregation (82.6 ± 0.86% after 24 h), positive exopolysaccharide production, milk fermentation capability with 21-day storage stability, and an antimicrobial activity against *Staphylococcus aureus* ATCC 25923, *Salmonella enterica* ATCC 14028, *Klebsiella pneumoniae* ATCC 10031, and *Escherichia coli* ATCC 25922. In vivo administration of *L. plantarum* Y_10b_ in a MetS rat model resulted in significant hypoglycemic, hypocholesterolemic, and anti-obesity effects. In conclusion, *L. plantarum* Y_10b_ is a promising probiotic for managing MetS manifestations. Further clinical investigations for use as a therapeutic intervention are highly recommended.

**Supplementary Information:**

The online version contains supplementary material available at 10.1007/s12602-025-10896-6.

## Introduction

Metabolic syndrome (MetS) is diagnosed if three or more of the following clinical markers are present: obesity, dyslipidemia, high blood pressure, and insulin resistance [1]. MetS is usually associated with an increased risk of cardiovascular diseases, stroke, and type 2 diabetes mellitus (T2DM) [2]. High prevalence rates have been recorded worldwide for MetS [3], and have increased significantly over time among US adults, from 28.23% in 1999 to 37.09% in 2018 [4].

Lifestyle modification and dietary therapies are the first-line treatments for MetS. Medicines are also often prescribed to treat the different medical conditions associated with MetS, such as hypertension, dyslipidemia, obesity, and hyperglycemia. Unfortunately, there will never be a medication without adverse effects. Additionally, the use of a combination of medications to treat the various associated conditions can lead to drug interactions and reduced patient compliance [5, 6].

Probiotics are defined as living microorganisms that, when administered in adequate amounts, confer health benefits to the host [7]. Lactic acid bacteria (LAB) are Gram-positive, non-pathogenic, and a major group of probiotics that have been generally recognized as safe. *Lactobacillus* is the largest genus in this group, besides other genera, such as *Lactococcus*, *Pediococcus*, *Leuconostoc*, *Streptococcus*, and *Enterococcus* [8]. Different health benefits have been reported for LAB probiotics, including competing with gut pathogens for binding sites and nutrients, producing antimicrobials like bacteriocins and short-chain fatty acids, enhancing the immune system’s response, and modulating the gut microbiota. Additionally, some species exhibit hypocholesterolemic and hypoglycemic effects, with potential applications in metabolic disorders, such as T2DM and hypercholesterolemia [9]; these effects are usually strain- and disease-specific [10]. *Lactiplantibacillus plantarum* has demonstrated antioxidant and antimicrobial activities [11]. *Lactococcus lactis* has many proven health benefits, including antimicrobial activity, maintenance of intestinal integrity, and anticarcinogenic effects [12]. Additionally, other LAB species have different beneficial properties, such as *Streptococcus thermophilus*, which has many benefits for alleviating gastrointestinal disorders due to its anti-inflammatory and antimicrobial effects, and for maintaining the gut microbiome balance. Also, *Pediococcus pentosaceus* has numerous health benefits, including antioxidant, antimicrobial, cholesterol-lowering, and immune-modulation effects [13, 14].

Fermented traditional Egyptian foods are rich in microbial diversity with unexplored activities, from which probiotic LAB have already been isolated [15, 16]. Different *Lactobacillus* species were isolated from traditional fermented milk (Laban rayeb) [17]. Also, many LAB were isolated from Domiati cheese (Gbnah beeda) as *Leuconostoc mesenteroides*,* Lactobacillus versmoldensis*, and *Lactococcus lactis* [18]. In addition, *Enterococcus faecium*, *Enterococcus lactis*,* Enterococcus massiliensis*, and *L. plantarum* were isolated from different Egyptian fermented foods, including soy milk, kareish cheese, and plant-based luncheon. Promising strains require extensive screening and evaluation in vitro and in vivo*.*

In this study, probiotic LAB from traditional Egyptian foods and juices were evaluated for their capacity to modulate the manifestations of MetS. Many studies from Egypt have reported the in vitro beneficial effects of probiotics on health, such as antimicrobial, anticancer, antioxidant, hypocholesterolemic, and hypoglycemic effects [16, 19]. However, our work provides an integrated approach combining in vitro probiotic characterization with in vivo evaluation in a MetS model, highlighting the functional and therapeutic potential of locally isolated LAB strains.

## Methods

### Isolation, Identification, and Storage of Lactic Acid Bacteria

Samples, from local vendors (*n* = 12), were collected from typical Egyptian foods and juices (apple molasses, boza, cane juice, capucha lettuce, cottage cheese, herring, kishk, mesh, milk, pomegranate molasses, rayeb, and yogurt) in clean sterile containers and transported immediately to the lab, on ice. The collected samples were used to inoculate deMan, Rogosa, and Sharpe (MRS) broth (10%; Himedia, India). The inoculated broth was incubated at 37 °C for 48 h under anaerobic conditions, using a candle jar, followed by serial dilution in phosphate-buffered saline (PBS), spreading on MRS agar (Himedia, India), and incubation under anaerobic conditions at 37 °C for 48 h. Morphologically distinct colonies were selected and characterized by Gram-staining and catalase activity, where LAB displayed the typical Gram-positive (rod-shaped or coccoid) and catalase-negative phenotypes [20].

LAB isolates were identified by 16S rRNA sequencing or Matrix-Assisted Laser Desorption Ionization-Time of Flight Mass Spectrometry (MALDI-TOF MS; VITEK MS system, BioMérieux, France). For 16S rRNA sequencing, a fresh colony of the isolated LAB was subjected to genomic DNA extraction using the PrepMan Ultra sample preparation kit (ThermoFisher, USA), according to the manufacturer’s instructions. The 16S rRNA gene was amplified using the universal primers: 27 F (5’-AGAGTTTGATCMTGGCTCAG-3’) and 1492R (5’ TACGGYTACCTTGTTACGACTT-3’) [21]. The PCR products were purified using QIAquick PCR Purification Kit (Qiagen, Hilden, Germany), according to the manufacturer’s instructions, and sequenced using the Sanger sequencing method. The resulting sequences were subjected to a homology search using the NCBI BLASTn tool (https://blast.ncbi.nlm.nih.gov/Blast.cgi). The sequence of the isolate, with promising glucose- and cholesterol-lowering levels in vitro, was deposited in GenBank.

LAB isolates were stored in MRS broth containing 20% (v/v) glycerol at −20^°^C [16]. When needed, cultures from glycerol stock were isolated on MRS agar and incubated under anaerobic conditions for 48 h at 37 °C.

The promising isolate was lyophilized using a freeze-drying system (model BK-FD12S) at −50 °C under vacuum for 24 h. The viable cell count of the lyophilizate was determined. The lyophilizate was distributed into Eppendorf tubes and stored at −20 °C until use. When needed, the lyophilized powder was suspended in PBS to achieve the desired colony-forming unit (CFU) count, immediately before use [22].

### In Vitro Screening of Lactic Acid Bacteria Isolates for Glucose- and Cholesterol-Lowering Potential

The LAB isolates were evaluated for in vitro glucose- and cholesterol-lowering potential. The isolate with the most promising result was selected for further testing.

#### In Vitro Glucose-Lowering Activity

A Fresh overnight culture of the tested isolate in MRS broth was diluted in MRS broth containing 20% glucose to achieve an optical density at 600 nm (OD_600_) of 1. The OD-adjusted cultures were incubated at 37 °C under anaerobic conditions, and glucose levels were measured at 0, 6, and 12 h using a blood glucometer (Element, Korea) [23]. The percentage reduction in glucose concentration was calculated as follows: Reduction (%) = (C_0_- C_1_/C_0_)× 100, where C_0_ was the glucose concentration at time zero, and C_1_ was the glucose concentration at different time intervals. The experiment was performed in triplicate.

#### Cholesterol Assimilation Testing

Freshly isolated colonies were cultured in MRS broth supplemented with 0.3% ox gall (Loba Chemie, India) and 0.1 g/L water-soluble cholesterol (Sigma-Aldrich, USA), and incubated under anaerobic conditions at 37 °C for 48 h. An uninoculated negative control consisting of MRS broth supplemented with the same concentrations of ox gall and cholesterol was included. At the end of the incubation period, cells were removed by centrifugation at 9000 rpm for 15 min at 4 °C, and cholesterol levels were measured in the cell-free supernatant (CFS) [24]. The percentage reduction in cholesterol level was calculated as follows: Reduction (%) = (1- A_1_/A_0_)× 100, where A_1_ was the absorbance of the test, and A_0_ was the absorbance of the control. The experiment was performed in triplicate.

#### Bile Salt Hydrolase Production

Bile salt hydrolase (BSH) production was assessed using MRS agar plates supplemented with 0.5% bile salt mixture of cholic and deoxycholic acid sodium salts (Merck, Germany). Wells measuring 7 mm in diameter were cut in the plates and inoculated with 200 µL of a fresh overnight culture of LAB in MRS broth (OD_600_ of 1). Plates were incubated under anaerobic conditions for 48 h at 37 °C. The diameter of the precipitation zone around each well, indicating BSH production, was measured and recorded [25]. The experiment was performed in triplicate.

### Assessment of the Probiotic Characteristics of the Selected Isolate

#### Tolerance to Simulated Gastrointestinal Conditions

The test was carried out according to Zhang and colleagues [26], with some modifications. An artificial gastric juice was freshly prepared by suspending 3 g/L pepsin 1:3000 (Loba Chemie, India) in PBS, and the pH was adjusted to 3 with 1 N HCl. An artificial intestinal juice was freshly prepared by suspending 2 g/L trypsin (2000 U/g, Loba Chemie, India) and 0.3% bile salts (Loba Chemie, India) in PBS, and the pH was adjusted to 8 with 1 N NaOH.

Cells from a 3 mL overnight culture of the tested isolate in MRS broth were harvested by centrifugation at 5000 rpm for 10 min at 4 °C. The pellet was washed three times with PBS and resuspended in 3 mL of the artificial gastric juice (0 h_g_), followed by incubation at 37 °C for 2 h (2 h_g_). Cells were then harvested by centrifugation, washed with PBS and resuspended in 3 mL of artificial intestinal juice (0 h_i_), followed by incubation at 37 °C for 8 h (8 h_i_). The viable count was determined at 0 h_g_, 2 h_g_, 0 h_i_, and 8 h_i_. The experiment was performed in triplicate.

#### Cell Surface Hydrophobicity

The cell surface hydrophobicity (CSH) was assessed by measuring the affinity of bacterial cells for hydrocarbons. Briefly, the bacterial pellet from a fresh overnight culture in MRS broth was washed and resuspended in 5 mL PBS. The absorbance of the resulting suspension was measured at 600 nm (A_0_). Then, 3 mL of the prepared suspension was added to 1 mL of chloroform, vortexed for 1 min, and incubated at 37 °C for 30 min without shaking to allow separation of the organic and aqueous phases. Carefully, 1 mL of the upper aqueous phase was removed, and its absorbance was measured at 600 nm (A_1_). The hydrophobicity (%) was calculated as follows: Hydrophobicity (%) = (1-A_1_/A_0_) × 100 [27]. The experiment was performed in triplicate.

#### Auto-aggregation

The auto-aggregation capacity of the selected isolate was evaluated by suspending the bacterial pellet in PBS, as described under CSH. The absorbance of the upper portion was measured at different time intervals (0, 3, and 24 h). The auto-aggregation percentage was calculated as follows: Auto-aggregation (%) = (1-A_1_/A_0_) × 100, where A_0_ was the absorbance at time zero and A_1_ was the absorbance at different time intervals [28]. The experiment was performed in triplicate.

#### Milk Fermentation and Maintenance of Functional Properties during Storage in Milk

The ability of LAB to ferment milk and survive in fermented milk is an important property, enabling their use in dairy products. The fermentation capability was assessed by inoculating 10% skimmed milk (Himedia, India) with 1% of a fresh overnight culture of the selected isolate in MRS broth, diluted to an OD_600_ of 1. The inoculated media were incubated at 37 °C for 24 h under anaerobic conditions. Fermented milk samples were stored at 4 °C for 21 days. Cell viability was assessed at 0 h, 24 h, and after 7, 14, and 21 days of storage, by determining the total microbial counts. The pH was monitored at 0 h, 7, 14, and 21 days [29]. The experiment was performed in triplicate.

The maintenance of the probiotic functional properties, glucose utilization, and cholesterol assimilation capabilities of the selected isolate during storage under refrigeration, was evaluated over 21 days. Samples were withdrawn from the fermented milk culture after 24 h at 37 °C, and after 7, 14, and 21 days of storage at 4 °C. The in vitro glucose-lowering activity after 12 h and cholesterol assimilation activity were determined at each time point, as previously described, using uninoculated skimmed milk (10% w/v) as a control.

The in vitro glucose-lowering and cholesterol assimilation activities in milk samples were compared to overnight cultures in MRS broth. The percentage reduction in glucose and cholesterol concentrations was normalized to the viable count of each sample. All experiments were performed in triplicate.

### Evaluation of Other Beneficial Characteristics of the Selected Isolate

#### Exopolysaccharide Production

The ability of the selected isolate to produce an exopolysaccharide (EPS) was assessed by culturing on MRS agar and incubating at 37 °C for 48 h under anaerobic conditions. Following incubation, a metal loop was used to touch and lift the colonies that were considered positive for EPS production if a streak was raised by more than 1.5 mm [30]. The experiment was performed in triplicate.

#### Antimicrobial Activity

The antimicrobial activity of the tested isolate was determined, using the agar-well diffusion method, against four food-borne pathogens: *Staphylococcus aureus* ATCC 25923, *Salmonella enterica* ATCC 14028, *Klebsiella pneumoniae* ATCC 10031, and *Escherichia coli* ATCC 25922. Briefly, a fresh overnight culture of the tested pathogens was prepared in tryptic soy broth (TSB; LabM, UK) and adjusted to 0.5 McFarland (approximately 1 × 10⁸ CFU/mL), and further diluted 1:20 in TSB to obtain a working inoculum of approximately 5 × 10⁶ CFU/mL. Seed inoculation of 100 µL of the adjusted culture was made in molten Muller-Hinton agar (10 ml; LabM, UK), and wells of 7 mm in diameter were cut in the inoculated agar plates. Then, 100 µL of the supernatant (sterilized by a 0.22 μm membrane filter) from a fresh overnight culture (CFS) of the selected LAB isolate in MRS broth was used to fill the wells. The plates were incubated for 24 h at 37 °C under aerobic conditions, and the diameters of the inhibition zones formed around the wells were measured [31]. The experiment was performed in triplicate.

##### Determination of the Possible Antimicrobial Mechanism

To determine whether the antimicrobial activity was due to the production of organic acids or other antimicrobial compounds, the CFS was divided into two aliquots. One aliquot was maintained at its original acidic pH (control), while the second aliquot was neutralized to pH 6.5 using 5 M NaOH, to eliminate the effect of the organic acids [31]. The antimicrobial activity of the neutralized and control CFS samples was determined against all four pathogens using the well-diffusion method, as previously described. The experiment was carried out in triplicate.

##### Determination of the Minimum Inhibitory Concentration

The minimum inhibitory concentration (MIC) of the CFS against the tested pathogens was determined using the broth microdilution method. Two-fold serial dilutions of the CFS in Muller-Hinton broth were prepared. Each well received 10 µL of the adjusted culture of the tested pathogens, resulting in a final inoculum of approximately 5 × 10⁵ CFU/mL. The plates were incubated at 37 °C for 24 h under aerobic conditions. Positive and negative controls were included. The MIC was defined as the lowest concentration (highest dilution) of CFS that completely inhibited visible bacterial growth [32]. The experiment was carried out in triplicate.

##### Determination of the Minimum Bactericidal Concentration

The minimum bactericidal concentration (MBC) was determined from wells showing no visible growth in the MIC assay. From each clear well, 10 µL aliquots were spotted onto Tryptic Soy Agar (TSA) plates. Plates were incubated at 37 °C for 24 h and examined for bacterial growth. The MBC was defined as the lowest concentration of CFS that resulted in ≥ 99.9% killing of the initial inoculum (no visible growth on agar plates) [33]. The experiment was carried out in triplicate. The mode of action (bacteriostatic vs. bactericidal) was determined by calculating the MBC/MIC ratio. An MBC/MIC ratio ≤ 4 indicates bactericidal activity, while a ratio > 4 suggests bacteriostatic activity [34].

##### Kinetic (time-kill) Assay

Based on the MIC and MBC values of the CFS against the tested pathogens, working solutions of CFS were prepared in TSB to achieve final dilutions of 1× MIC, 2× MIC, and 4× MIC. The bacterial inoculum was added to the working solutions to obtain a final bacterial concentration of approximately 10^5^ CFU/mL. All tubes were incubated aerobically at 37 °C, and aliquots of 100 µL were aseptically withdrawn from each tube for viable count determination at 0, 3, 6, 12, and 24 h. The CFS was considered bactericidal if it caused ≥ 3 log₁₀ reduction in viable count compared to the initial inoculum, while reductions < 3 log₁₀ were considered bacteriostatic [35].

### In Vivo Evaluation of the Efficiency of the Probiotic Strain in Alleviating Metabolic Syndrome Manifestation

The efficacy of the selected LAB isolate in alleviating the manifestations of MetS was evaluated in 6-week-old Wistar rats (*n* = 28) weighing 170–200 g [36, 37]. All procedures and interventions were approved by the Ethics Committee of the Faculty of Pharmacy, Cairo University, Cairo, Egypt (Approval no. MI (2968)), following the guidelines established by the National Research Council Guide for the Care and Use of Laboratory Animals [38]. The rats were housed in individual cages under controlled temperatures (22 ± 2 °C) with a 12 h light/dark cycle and were acclimatized for one week before the experiment. The animals had free access to food and water throughout the experiment, except when fasting blood glucose (FBG) was to be measured; in such a case, food was withheld for 8 h prior to testing.

Fasting blood glucose (FBG) levels were measured in the blood collected from the rats’ tails using a blood glucometer (Element, Korea) at the beginning of the experiment to ensure that none of the rats were diabetic (FBG < 150 mg/dL [39]). The Lee index (a measure of obesity in rodents) was also calculated at the beginning of the experiment to ensure that none of the rats were obese (Lee index ≤ 310 [40]). Lee index was calculated using the following formulae: Lee index = (∛Body weight​)/Naso-anal length × 1000, where the weight was measured in grams, and the naso-anal length was measured in centimeters [40].

#### Induction of Hypercholesterolemia

The rats were divided into four groups, each consisting of seven rats. The MetS groups (I and II) received a high-fat diet (HFD) for 4 weeks to induce hypercholesterolemia [36]. The HFD consisted of the following per kilogram: 10 g of cholesterol (Loba Chemie, India), 63 g of vitamins, minerals, and amino acids (AD3E Victoir, Egypt), 250 g of casein (Optimum Nutrition, UK), 310 g of beef lard, 1 g sodium chloride, 1 g dried yeast, and 365 g of the normal pellet diet (ND; Alfa Feed, Egypt) [41]. The control groups (III and IV) received only the ND. FBG levels were measured after 4 weeks of receiving the assigned diet, while the Lee index was measured weekly till the end of the experiment.

#### Induction of Type 2 Diabetes Mellitus

T2DM was induced 4 weeks after the HFD feeding in groups I and II by streptozotocin (STZ) injection. The MetS groups (I and II; HFD-fed) were injected intraperitoneally with 35 mg/kg body weight of freshly prepared STZ in citrate buffer [37, 42], and were given 10% glucose in their drinking water for 24 h before returning to normal drinking water [42]. Seventy-two hours after STZ injection, the FBG level was measured to confirm the development of T2DM (FBG > 150 mg/dL). Groups III and IV (ND-fed) were injected intraperitoneally with citrate buffer as a control and provided with normal drinking water.

#### Probiotic Administration

After successful induction of T2DM (FBG > 150 mg/dL, 72 h post-STZ administration), a dose equivalent to 1 × 10^9^ CFU/kg body weight of the lyophilized LAB strain suspended in saline was administered daily to group I (HFD-fed MetS group) and group III (ND-fed control group) by oral gavage, using 18-gauge rounded/bulb-tipped gavage, for 4 weeks [43]. The remaining two groups received normal saline as a control (Online resource 1, Supplementary Fig. 1). During the entire probiotic administration period, the MetS groups (I and II) received HFD while the control groups (III and IV) received ND.

#### Evaluation of the Hypoglycemic and Hypocholesterolemic Effects of Probiotic Administration

The hypoglycemic and hypocholesterolemic effects of probiotic administration were evaluated by measuring FBG, the Lee index, and total cholesterol levels. The FBG level and Lee index were determined weekly throughout the probiotic treatment period, as described previously. At the end of the 4-week treatment period, blood samples (1 mL) were collected from the retro-orbital plexus of the rats’ eyes, under anesthesia with isoflurane 2.5% (Merck, Germany), using a capillary tube; the collected blood was transferred into yellow-capped tubes containing a clot activator and gel separator. The samples were allowed to clot at room temperature for 30 min and then centrifuged at 6000 rpm for 10 min at 4 °C to separate the serum [44]. The total cholesterol level was determined using an enzymatic colorimetric method on a Cobas C311 chemistry analyzer (Roche Diagnostics, Germany), using the Cholesterol Gen-2 reagent (Roche Diagnostics, Germany) [45].

### Statistical Analysis

The statistical analysis was performed using GraphPad Prism version 9 (GraphPad Software, California, USA). The milk fermentation viable count experiment results were analyzed using one-way analysis of variance (ANOVA) followed by Dunnett’s post hoc analysis. The results of in vivo blood cholesterol level and normalized percentages of in vitro glucose- and cholesterol-lowering activities after storage in milk were analyzed using one-way ANOVA followed by Tukey’s post hoc analysis. Comparisons between multiple groups in the remaining experiments were performed using two-way ANOVA, with Tukey’s multiple comparison test for post-hoc analysis. The correlation between CSH and auto-aggregation was evaluated using the Pearson correlation coefficient (r) to determine the linear relationship between these variables. Statistical significance was defined as *p* < 0.05.

## Results

### Isolation of Lactic Acid Bacteria from Egyptian Cuisine

Out of 12 Egyptian cuisine food samples, 42 morphologically distinct bacterial isolates were recovered, including 10 isolates that displayed the typical Gram-positive (rod-shaped or coccoid) and catalase-negative phenotypes associated with LAB species. The isolates, their sources, and identification are provided in Table 1.

**Table 1:** Probiotic lactic acid bacteria recovered from different Egyptian foods and juices

Isolate code	Source	Identification
Y_3_	Boza	*Leuconostoc citreum*
Y_4_	Yogurt	*Streptococcus thermophiles*
Y_5b_	Herring	*Lactobacillus sakei*
Y_5c_	Herring	*Enterococcus faecium*
Y_6_	Capucha-Lettuce	*Enterococcus hirae*
Y_7_	Rayeb	*Lactococcus Lactis*
Y_8_	Cane juice	*Limosilactobacillus fermentum*
Y_9_	Milk	*Pediococcus pentosaceus*
Y_10a_	Cottage cheese	*Enterococcus faecium*
Y_10b_	Cottage cheese	*Lactiplantibacillus plantarum*

### *Lactiplantibacillus Plantarum* Y_10b_ Isolate Exhibited the Optimum Glucose and Cholesterol Utilization Activities In Vitro

**Fig. 1:**
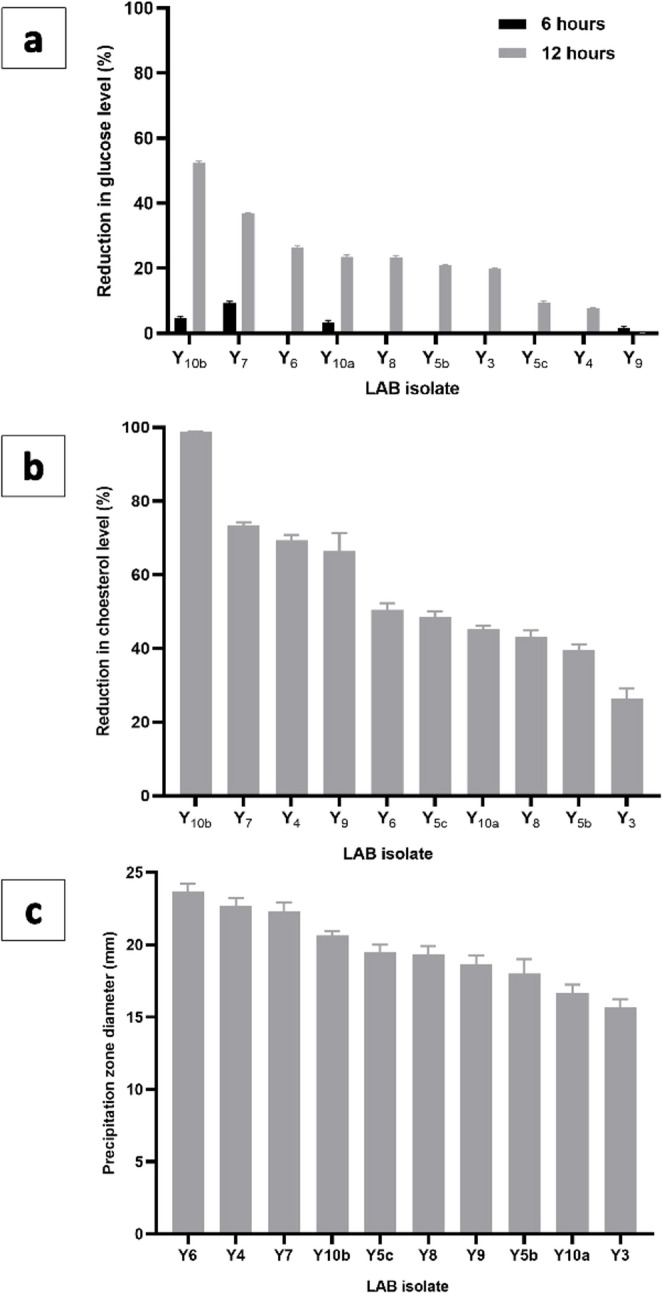
In vitro glucose- and cholesterol-lowering activities of the isolated lactic acid bacteria (LAB). **(a)** Reduction in glucose level by LAB isolates measured after 6 and 12 h of incubation of the tested isolate in glucose-containing deMan, Rogosa, and Sharpe (MRS) broth; **(b)** Reduction in cholesterol level after 48 h of incubation in MRS broth containing ox gall and cholesterol; **(c)** The precipitation zone diameters formed around wells filled with MRS broth cultures of LAB isolates in bile salt mixture-containing MRS agar plates after incubation for 48 h, as an indication of the bile salt hydrolase activity

All the tested isolates, except Y_9_, showed a significant reduction in glucose level, with isolate *L. plantarum* Y_10b_ exhibiting the highest level of glucose reduction (53.4 ± 0.47%) after a 12-hour incubation period in 20% glucose (Fig. 1a). Similarly, all the tested isolates exhibited a significant reduction in cholesterol levels compared to the control, with isolate *L. plantarum* Y_10b_ recording the highest cholesterol assimilation, resulting in a 98.69 ± 0.18% reduction in cholesterol levels (Fig. 1b). Additionally, isolate *L. plantarum* Y_10b_ was among the top four isolates with the highest BSH production, indicated by the diameter of the precipitation zone formed on bile salt-supplemented agar plates (Fig. 1c). Therefore, *L. plantarum* Y_10b_ was selected for further testing. The 16S rRNA amplified gene sequence of Y_10b_ showed 100% identity to *Lactiplantibacillus plantarum* strain CIP 103151 (Accession no. NR_104573.1; Table 1) and was deposited in GenBank under accession no. PQ516964.

### Probiotic Characteristics of *L. Plantarum* Y_10b_ Isolate

The *L. plantarum* Y_10b_ isolate exhibited a good tolerance to simulated gastrointestinal juice, where no significant reduction in viable microbial count was recorded after 2 h of exposure to simulated gastric juice and 8 h of exposure to simulated intestinal juice (0.07 and 0.08 log_10_ CFU/mL reduction in counts, respectively). *L. plantarum* Y_10b_ isolate also showed 70.29 ± 0.78% hydrophobicity to chloroform after 30 min of exposure, a measure of cell surface hydrophobicity. Additionally, *L. plantarum* Y_10b_ exhibited a time-dependent increase in auto-aggregation (52.1 ± 0.75% and 82.6 ± 0.86% at 3 h and 24 h). A positive correlation was observed between the CSH and the auto-aggregation of *L. plantarum* Y_10b_ (*r* = 0.5532).

### Extra Beneficial Characteristics of *L. Plantarum* Y_10b_ Isolate

#### Exopolysaccharide Production

*L*. *plantarum* Y_10b_ isolate produced slimy colonies on MRS agar plates, indicating EPS production.

#### Milk Fermentation Capability and Maintenance of Functional Properties during the Refrigerated Storage of Fermented Milk

**Fig. 2:**
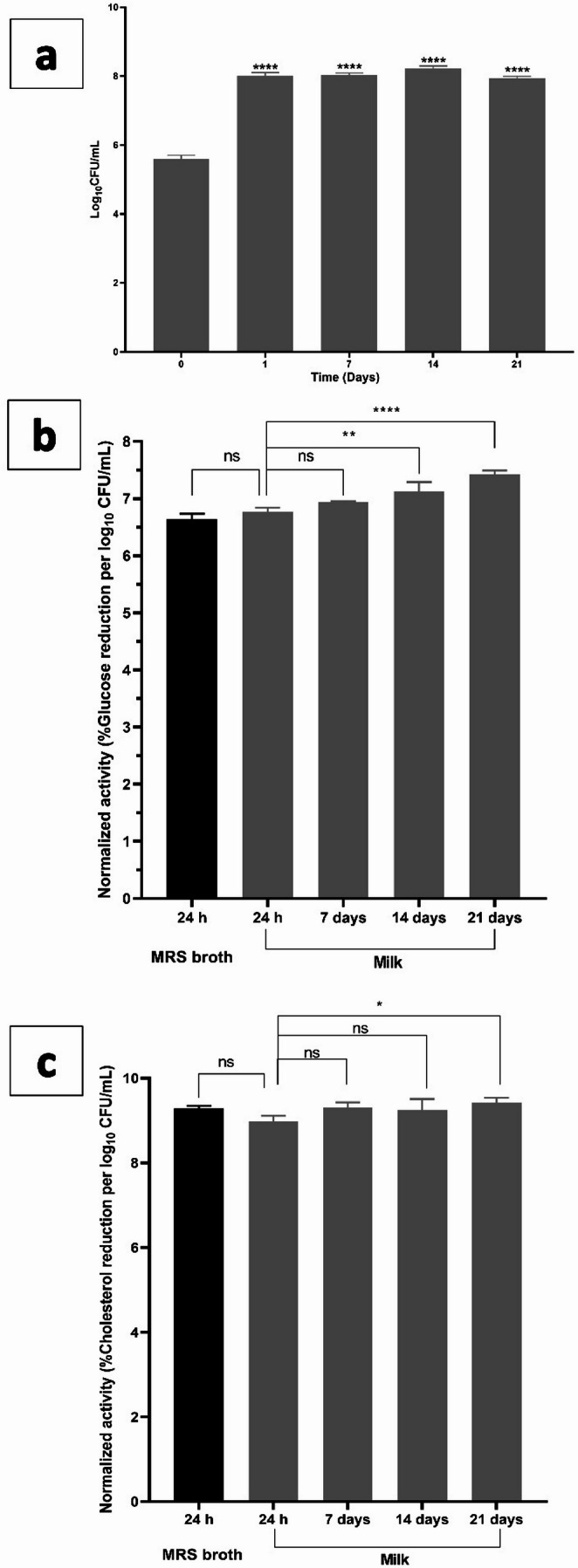
Milk fermentation capability and functional stability of *Lactiplantibacillus plantarum* Y_10b_ isolate during storage. *L. plantarum* Y_10b_ was incubated in skimmed milk at 37 °C for 24 h, followed by storage in the refrigerator at 4 °C for 21 days. Samples were withdrawn from milk, and **(a)** viable counts were determined at 0 time and after incubation for 1 day at 37 °C and after storage at 4 °C for 7, 14, and 21 days. The statistical significance of the viable count at different time points compared to zero time was determined by one-way ANOVA followed by Dunnett’s post-hoc test. The maintenance of **(b)** glucose- and **(c)** cholesterol-lowering activities of *L. plantarum* Y_10b_ during refrigerator storage in milk was determined by measuring the in vitro glucose-lowering activity after 12 h and cholesterol-assimilation activity from samples withdrawn after 7, 14, and 21 days of storage. The percentage reductions in glucose and cholesterol levels were normalized to the viable counts of their respective samples and compared with results obtained using a fresh culture of *L. plantarum* Y_10b_ in MRS broth. The statistical significance was determined by one-way ANOVA followed by Tukey’s post-hoc test. Asterisk refers to statistically significant difference as follows: * *p* < 0.05, ** refers to *p* < 0.01, **** refers to *p* < 0.0001, and ns for nonsignificance

*L. plantarum* Y_10b_ isolate successfully fermented milk, with a significant increase in cell counts observed after 24 h of incubation at 37 °C, in skimmed milk at a pH of 6.64 (*p* < 0.0001). Storage in skimmed milk at 4 °C maintained the count at 1.37 × 10^8^ ± 0.19 (Fig. 2a), with the pH dropping to 4.34 ± 0.17 after 7 days; the low pH was maintained throughout the 21-day storage period. *L. plantarum* Y_10b_ isolate retained the functional properties during storage at 4 °C. Glucose utilization capacity of *L. plantarum* Y_10b_ isolate in refrigerated fermented milk was maintained throughout the storage period, with a significant increase in activity, compared to fresh isolated colonies in MRS broth, after 14 and 21 days (*p* < 0.01 and *p* < 0.0001; Fig. 2b). Similarly, cholesterol assimilation capability was maintained throughout storage, with a significant increase in activity compared to fresh isolated colonies in MRS broth, after 21 days (*p* < 0.05; Fig. 2c).

#### Antimicrobial Activity

*L. plantarum* Y_10b_ isolate also possessed an antimicrobial activity against *S. aureus* ATCC 25923, *S. enterica* ATCC 14028, *K. pneumoniae* ATCC 10031, and *E. coli* ATCC 25922, with inhibition zone diameters of 16.7 ± 0.19 mm, 20 ± 0 mm, 17.6 ± 0.41 mm, and 17 ± 0 mm, respectively. Neutralizing the acidity of *L. plantarum* Y_10b_ CFS to pH 6.5 resulted in the complete loss of its antimicrobial activity against all tested bacteria, with no zones of inhibition observed. This demonstrates that the antimicrobial activity can be primarily mediated by the production of organic acids rather than by pH-independent antimicrobial compounds. The MIC of *L. plantarum* Y_10b_ CFS varied among the tested pathogens (Table 2), with a bactericidal activity against all tested strains.

**Table 2:** MIC and MBC values of *Lactiplantibacillus plantarum* Y_10b_ CFS against the tested pathogens

Pathogen	MIC (Dilution percentage)	MBC (Dilution percentage)	MBC/MIC Ratio	Mode of Action
*Staphylococcus aureus* ATCC 25923	25% ± 0%	25% ± 0%	1	Bactericidal
*Klebsiella pneumoniae* ATCC 10031	12.5% ± 0%	25% ± 0%	2	Bactericidal
*Salmonella enterica* ATCC 14028	12.5% ± 0%	25% ± 0%	2	Bactericidal
*Escherichia coli* ATCC 25922	12.5% ± 0%	25% ± 0%	2	Bactericidal

**Fig. 3:**
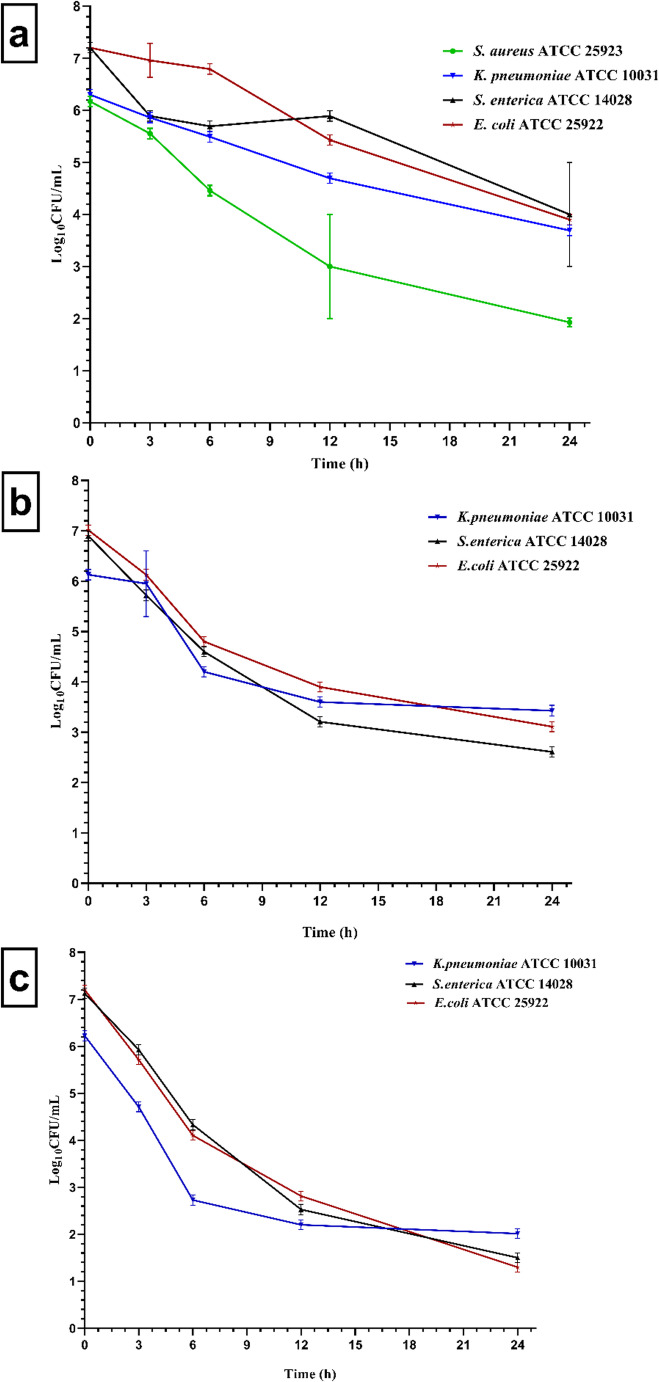
Time-kill curves showing the bactericidal activity of *Lactiplantibacillus plantarum* Y_10b_ cell-free supernatant (CFS) against the tested pathogens. *Staphylococcus aureus* ATCC 25923, *Klebsiella. pneumoniae* ATCC 10031, *Salmonella enterica* ATCC 14028, and *Escherichia coli* ATCC 25922 were exposed to CFS at **(a)** 1× MIC, **(b)** 2× MIC, and **(c)** 4× MIC concentrations. Viable counts (log₁₀ CFU/mL) were determined at 0, 3, 6, 12, and 24 h

In the time-kill assay, *L. plantarum* Y_10b_ CFS exhibited bactericidal activity against all tested pathogens and at all CFS dilutions, after 24 h (Fig. 3).

### *Lactiplantibacillus Plantarum* Y_10b_ Administration Alleviates Metabolic Syndrome Manifestations In Vivo

**Fig. 4:**
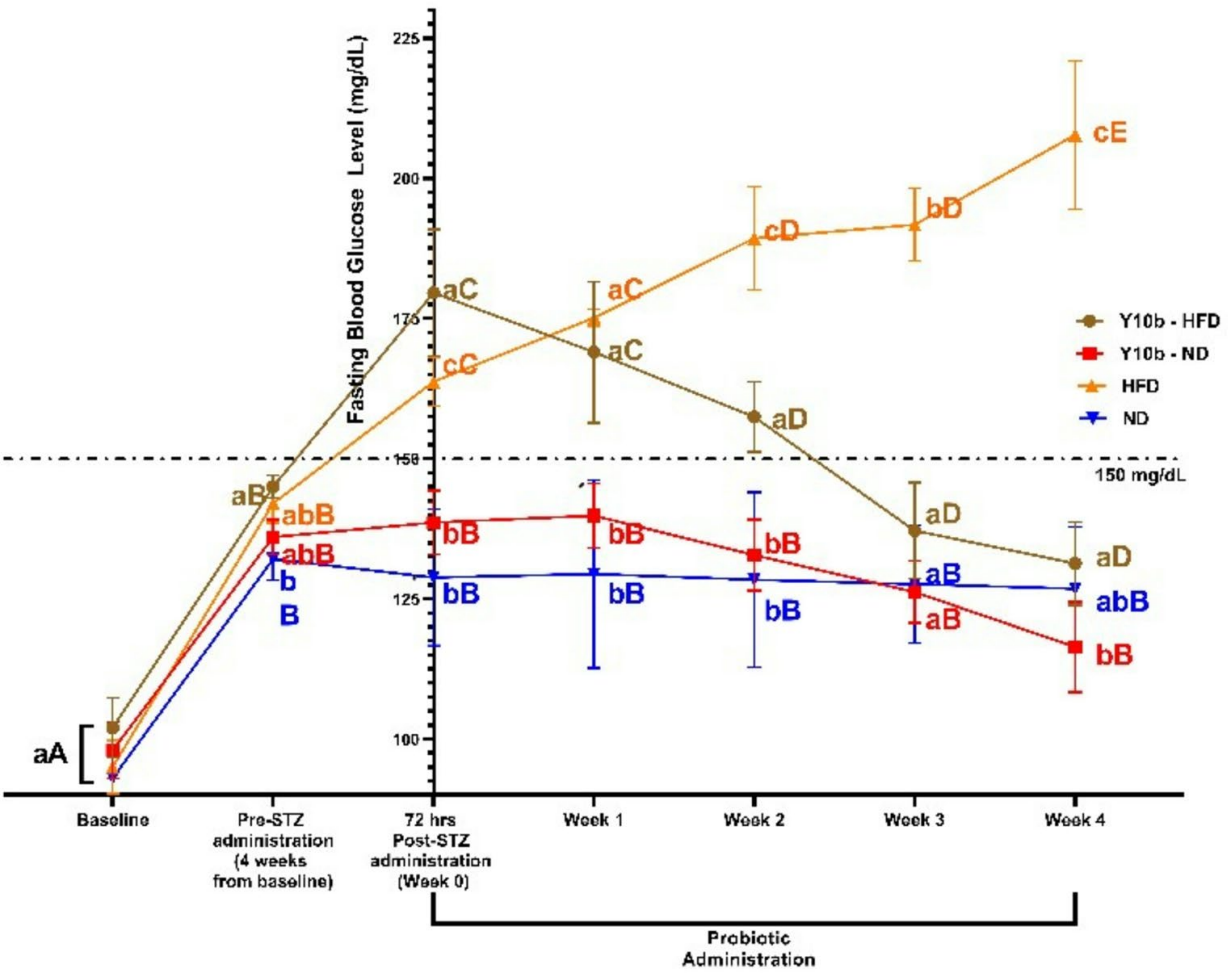
The hypoglycemic effect of *Lactiplantibacillus plantarum* Y_10b_ administration in Wistar rats with metabolic syndrome. The metabolic syndrome (MetS) was induced in Wistar rats by a high-fat diet (HFD) feeding for 4 weeks, followed by streptozotocin (STZ) injection. The fasting blood glucose level (FBG) was monitored at the baseline (before the beginning of HFD feeding), directly before STZ injection, and after 72 h of STZ administration to ensure the development of type 2 diabetes mellitus (FBG ˃ 150 mg/dL). *L. plantarum* Y_10b_ probiotic treatment was administered by oral gavage daily for 4 weeks, and the FBG was monitored weekly. The control MetS group on HFD received saline instead of the probiotic. Two other healthy control groups on a normal diet (ND) received *L. plantarum* Y_10b_ or saline. The midline represents the cut-off value for diabetic rats (150 mg/dL). A Two-way ANOVA with Tukey’s post-hoc test was used for multiple comparisons between the different groups at the same time point, and within the same group over different time points. Different lowercase letters (a, b) indicate significant differences between groups at the same time point, while different uppercase letters (A, B, C) indicate significant differences between time points within the same group (*p* < 0.05)

**Fig. 5:**
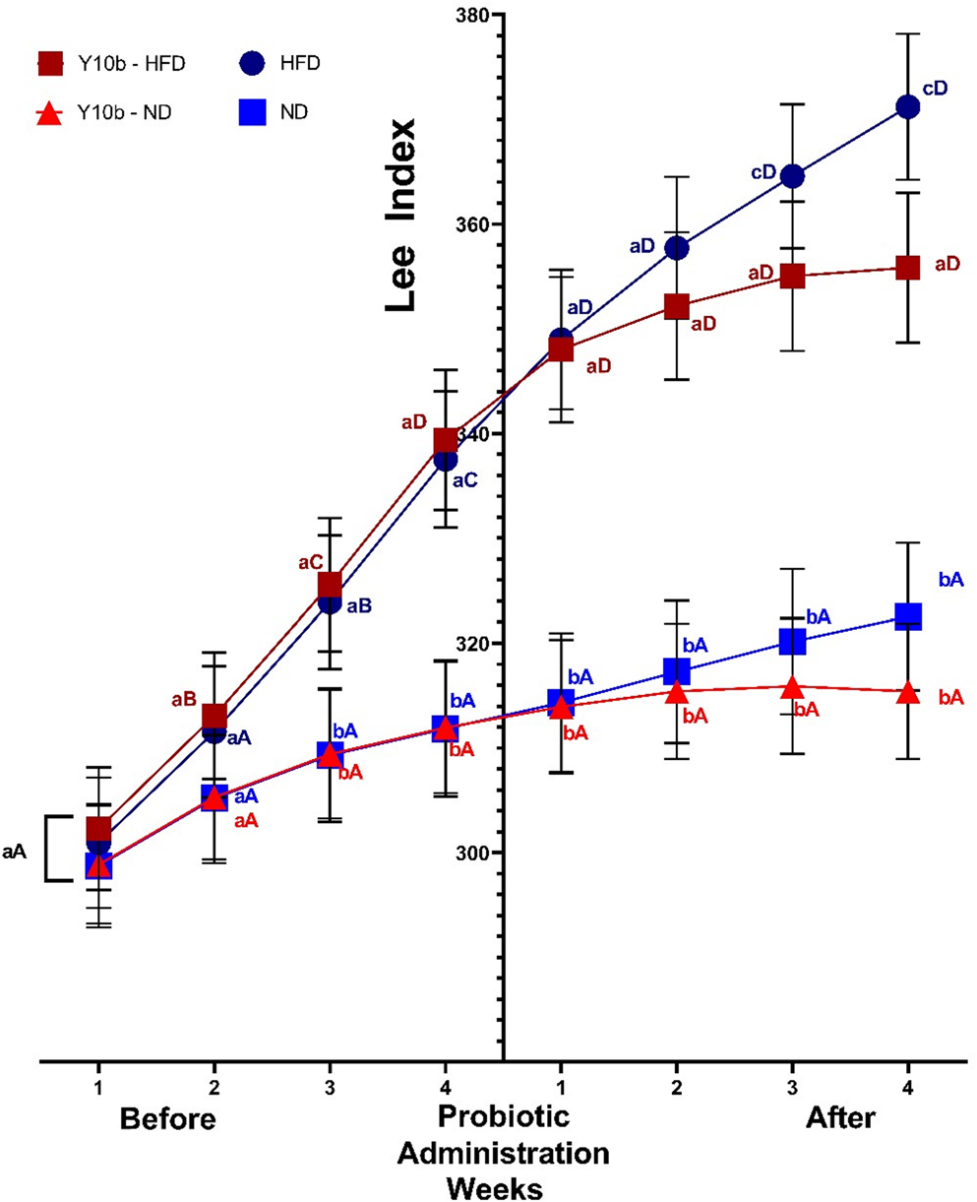
The obesity-reducing effect of *Lactiplantibacillus plantarum* Y_10b_ treatment in Wistar rats with metabolic syndrome. Obesity was induced in Wistar rats by a high-fat diet (HFD) feeding for 4 weeks. The induced obesity was assessed weekly by calculating the Lee index. *L. plantarum* Y_10b_ treatment was administered daily by oral gavage for 4 weeks, and its effect in reducing obesity was monitored by calculating the Lee index weekly. Control mice on HFD treated with saline daily were included. Additionally, two control groups on a normal diet (ND) receiving either *L. plantarum* Y_10b_ or saline were also included. Different lowercase letters (a, b) indicate significant differences between the groups at the same time point, while different uppercase letters (A, B, C) indicate significant differences between time points within the same group (*p* < 0.05)

The hypoglycemic and hypocholesterolemic effects of *L. plantarum* Y_10b_ isolate were evaluated employing a MetS model in male Wistar rats. The MetS groups (I and II) received HFD throughout the whole experiment, while the control groups (III and IV) received ND. The FBG level of all rats (on HFD and ND) was < 150 mg/dL (Mean = 138.6 ± 5.1 mg/dL), after 4 weeks of feeding the respective diet (Fig. 4). The rats on HFD were injected with a low dose of STZ to induce T2DM, where their FBG was > 150 mg/dL (Mean = 171.6 ± 11.2 mg/dL), after 72 h from STZ injection. However, the FBG of rats on ND and administered citrate buffer as a control was < 150 mg/dL (Mean = 133.7 ± 10.7 mg/dL) (Fig. 4). After the first 4 weeks of HFD feeding, all rats on HFD recorded a significantly elevated Lee indices (Mean = 338.5 ± 0.9) compared to those on ND (Mean = 311.9 ± 0.05; *p* < 0.05) (Fig. 5).

*L. plantarum* Y_10b_ isolate was administered daily to rats in groups I (MetS group) and III (control group), for 4 weeks, and the FBG and Lee index were monitored weekly, while the blood cholesterol level was determined at the end of the 4-week treatment period.

#### Blood Glucose Level

A significant reduction in FBG levels of the MetS group treated with *L. plantarum* Y_10b_ was recorded starting from the 2nd week of treatment (*p* < 0.0001) and continued throughout the 3rd and 4th week of treatment (*p* < 0.0001). By the end of the treatment period, the FBG in the MetS group treated with *L. plantarum* Y_10b_ was comparable to that of the control (ND) group. The untreated MetS group showed a significant increase in FBG by the 2nd week (*p* < 0.001) and continued to increase in the 3rd and 4th week (*p* < 0.0001; Fig. 4).

#### Lee Index

The effect of *L. plantarum* Y_10b_ administration on obesity-related changes was assessed using the Lee index. Administration of *L. plantarum* Y_10b_ for 3 and 4 weeks caused a significant reduction in the Lee index of rats in the MetS group compared to the untreated rats on HFD (*p* < 0.05 and *p* < 0.0001, respectively; Fig. 5).

#### Total Cholesterol Level

**Fig. 6:**
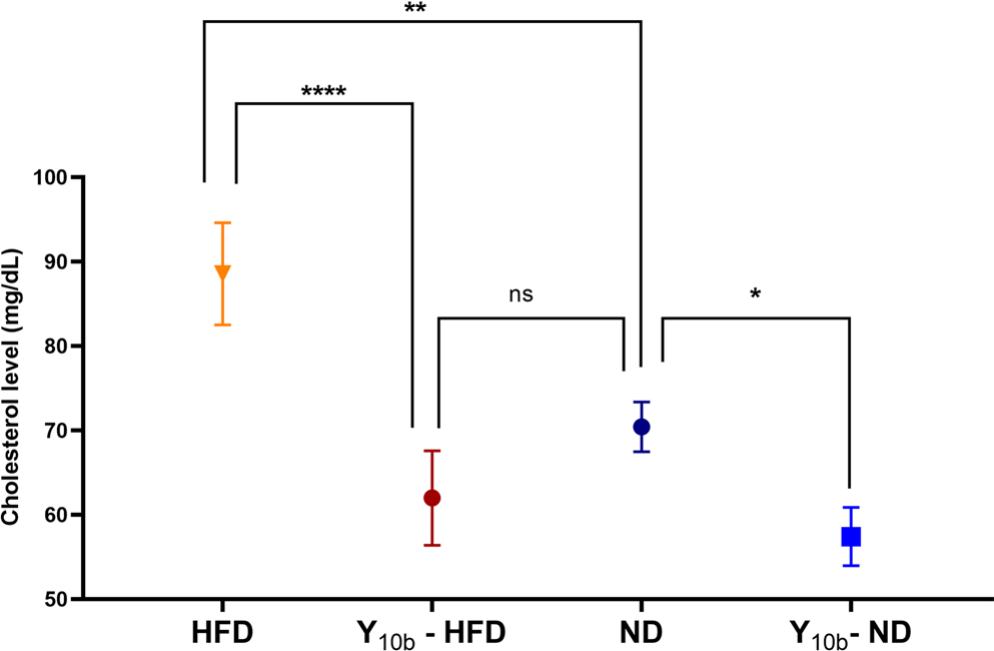
The hypocholesterolemic effect of *L. plantarum* Y_10b_ treatment in Wistar rats with metabolic syndrome. Metabolic syndrome (MetS) was induced in Wistar rats by high-fat diet (HFD) feeding for 4 weeks, followed by a streptozotocin injection. Rats were then treated with *L. plantarum* Y_10b_ daily via oral gavage for 4 weeks. Control mice on HFD, receiving saline daily, were included. Additionally, two control groups on a normal diet (ND) receiving either *L. plantarum* Y_10b_ or saline were also included. The total blood cholesterol level was determined at the end of the treatment period. One-way ANOVA followed by Tukey’s post-hoc test was used for multiple comparisons between the different groups. Asterisk refers to statistically significant difference as follows: *p < 0.05; **p < 0.01; ****p < 0.0001, and ‘ns’ for non-significance

Administration of the *L. plantarum* Y_10b_ isolate to rats with MetS for 4 weeks resulted in a significant reduction in blood cholesterol levels compared with the untreated group on HFD (*p* < 0.0001). The recorded blood cholesterol level in the treated MetS group was comparable to that of the control (ND) group (Fig. 6).

## Discussion

The rise in diet-related metabolic disorders, such as obesity, hypercholesterolemia, and T2DM, has prompted a growing interest in the use of probiotics as a natural and potentially effective intervention [46]. Among these, LAB has gained significant attention due to its long history of safe consumption and ability to modulate various physiological processes [8].

In this study, LAB isolates were recovered from diverse Egyptian foods and juice sources in search for a probiotic strain with potential benefits for alleviating MetS. In previous studies, many probiotic strains were isolated from the Egyptian cuisine with promising in vitro activities, such as *Lacticaseibacillus paracasei* BD3, *Lactiplantibacillus plantarum* BR4, and *Limosilactobacillus fermentum* MR2, which showed good bile salt hydrolase activity [30], and *Lactobacillus delbrueckii* subsp. *bulgaricus*,* Streptococcus thermophiles*, *Pediococcus acidilactici*, *Enterococcus mediterraneensis*,* Lactobacillus fermentum*, and *Streptococcus lutetiensis*, which exhibited antimicrobial activity [47, 48]. However, few studies have evaluated the health benefits of probiotic administration in vivo: antimicrobial activity in *H. pylori*-infected rats [49], hypocholesterolemic [50, 51], hypoglycemic [23], and wound healing effects [52].

The screening approach used in this study identified *L. plantarum* Y_10b_ as a promising strain with significant potential to regulate the MetS. Different *L. plantarum* strains isolated from Egyptian foods had various reported health-enhancement effects, including antibacterial activity against foodborne pathogens [53], antioxidant and anti-colon cancer activities [16]. The in vivo antibacterial [54], hypocholesterolemic [55] and neuroprotective [56] effects of *L. plantarum* strains isolated from Egyptian foods were also reported.

Initially, out of 42 isolates recovered from the Egyptian cuisine, 10 were phenotypically characterized as potential LAB isolates and underwent metabolic screening to assess their glucose- and cholesterol-lowering potential, as well as BSH activity.

Glucose consumption was evaluated in vitro, and *L. plantarum* Y_10b_ showed the highest glucose reduction percentage among the tested isolates. The ability of probiotics, including LAB, to reduce glucose levels through direct glucose consumption was previously reported [19, 23]. LAB use other mechanisms to reduce glucose levels, such as inhibiting α-glucosidase and α-amylase activities [57]. *L. plantarum* has been previously reported to exhibit an α-amylase inhibitory activity [58]. *L. plantarum* Y_10b_ produces EPS, which may contribute to reducing glucose levels, as α-glucosidase and α-amylase production may be inhibited through EPS production in LAB [59]. Modulation of glucose transporters, SGLT1 and GLUT2, is another mechanism by which different *Lactobacillus* spp., including *L. plantarum*, exert a hypoglycemic effect [60].

Lowering cholesterol levels is another important aspect in managing MetS. *L. plantarum* Y_10b_ exhibited the highest cholesterol reduction activity among the tested isolates. Several types of probiotics, including *L. plantarum* strains, have a cholesterol-lowering activity [61–63]. The cholesterol-lowering potential of probiotics can be attributed to several mechanisms, including cholesterol assimilation, production of metabolites that inhibit cholesterol synthesis or absorption, co-precipitation with deconjugated bile salts, and production of BSH [64, 65].

BSH is an enzyme that converts conjugated bile salts into deconjugated bile salts, which are less soluble and less efficiently reabsorbed in the intestine. Thus, increasing bile salt secretion in feces and reducing bile enterohepatic circulation, which accelerates bile acid synthesis using the cholesterol in the liver [66]. *L. plantarum* Y_10b_ was among the top isolates with high BSH activity. Different probiotic species have been reported to reduce cholesterol levels by utilizing the BSH activity [67, 68].

The probiotic properties of *L. plantarum* Y_10b_ were confirmed, as it showed good tolerance to simulated gastrointestinal passage. *L. plantarum* Y_10b_ maintained viability after 2 h of exposure to simulated gastric juice, suggesting an intrinsic mechanism for acid resistance, possibly including the ability to maintain intracellular pH homeostasis, proton-translocating ATPases, or the production of EPS that form a protective barrier [69]. It also tolerated simulated intestinal juice for 8 h. The resilience of *L. plantarum* Y_10b_ to intestinal juice could be attributed to the BSH activity, which deconjugates bile salts, thereby reducing their toxicity [70]. The microencapsulation of *L. plantarum* strains in a coating made of different polymers and oligosaccharides successfully enhanced their resistance to gastric conditions [71].

Another important characteristic of probiotics is the CSH, which facilitates attachment to host tissues [72], and enables the bacteria to persist for a prolonged period in the gut, thereby providing sustained benefits [73]. To be a good probiotic strain, the CSH is preferred to exceed 40% [74]; *L. plantarum* Y_10b_ exhibited significant hydrophobicity (70.29 ± 0.78%; *p* < 0.0001) after 30 min, compared to the zero-time control. Auto-aggregation also reflects the ability of probiotic strains to adhere to intestinal cells, facilitating colonization of the intestinal epithelium, biofilm formation, and modulation of gut microbiota [75]. *L. plantarum* Y_10b_ displayed time-dependent auto-aggregation. Additionally, a positive correlation was observed between CSH and auto-aggregation of *L. plantarum* Y_10b_ (*r* = 0.5532), as previously reported [76].

*L. plantarum* Y_10b_ also possessed many additional beneficial properties of probiotics, such as EPS production. The production of EPS is a desirable property for the food industry, serving as a natural bio-thickener, especially in the dairy sector, providing a desirable mouthfeel, creaminess, and firmness to food [77]. The EPS produced by the *L. plantarum* Y_10b_ isolate may enhance tolerance to gastrointestinal juices [78, 79], and exert a glucose-lowering effect by inhibiting α-glucosidase and α-amylase [59].

The milk-fermentation capability of probiotics is important for producing functional foods [80]. The significant increase in cell counts of *L. plantarum* Y_10b_ after incubation in skimmed milk for 24 h (*p* < 0.0001) at 37 °C indicates rapid growth in this medium. During the subsequent 21 days of storage at 4 °C in milk, *L. plantarum* Y_10b_ exhibited notable survival and remained metabolically active. The pH drop from 6.64 to 4.55 suggests acid production, a typical trait of LAB that can contribute to product preservation [81].

*L. plantarum* is highly versatile and can adapt to a wide range of food matrices. It preferentially grows and maintains its functional properties in nutrient-rich environments such as dairy-based fermented products (e.g., yogurt and cheese) and in various plant-based substrates (e.g., fermented vegetables, like sauerkraut, kimchi, and pickles). In addition, non-dairy beverages, including fruit and vegetable juices, have also been reported as suitable carriers, offering advantages for vegan consumers. These matrices not only support growth but also allow the strain to exhibit its probiotic activities, making them promising vehicles for future applications [82–84]. This is evidenced by the maintenance of the in vitro glucose-lowering and cholesterol assimilation activities for *L. plantarum* Y_10b_ stored refrigerated in fermented milk compared to fresh isolated colonies cultured in MRS broth.

The observed broad-spectrum antimicrobial activity of *L. plantarum* Y_10b_ against clinically significant bacterial strains, including *S. aureus*, *S. enterica*, *K. pneumoniae*, and *E. coli*, underscores its potential as a promising probiotic candidate. The antimicrobial activity of LAB is mediated through the production of various antimicrobial compounds, including organic acids, hydrogen peroxide, bacteriocins, and others [85]. In our study, neutralization of *L. plantarum* Y_10b_ CFS to pH 6.5 completely abolished the antibacterial activity, indicating that the inhibitory effect may be attributed to organic acids rather than bacteriocin-like compounds production. This finding is consistent with a previous study that reported that the antibacterial activity of the CFS from *L. plantarum* O7S1 was primarily attributable to organic acid production [86]. Different *Lactobacillus* spp. have been reported to inhibit biofilm formation by *Listeria monocytogenes* through different mechanisms [87]. The bactericidal activity of the CFS of LAB, including *L. plantarum* against foodborne pathogens has been previously reported [88].

Previous studies have highlighted *L. plantarum* as a LAB probiotic species [89–91]. Diverse strains of this species possess various health-promoting attributes, including anticancer, antioxidant, antimicrobial, and immune-boosting activities [91–93], as well as hypoglycemic and hypocholesterolemic effects [94].

The in vivo efficacy of *L. plantarum* Y_10b_ in alleviating the manifestations of MetS was confirmed in a Wistar rat model. The use of HFD in Wistar rats is a well-established method for inducing obesity and insulin resistance [36]. This dietary intervention simulates key features of MetS, characterized by progressive weight gain, altered lipid metabolism, and impaired insulin sensitivity. The 4-week duration allows sufficient time for significant metabolic alterations to manifest [95]. In our study, HFD-fed rats developed obesity after 4 weeks, evidenced by a consistent increase in the Lee index above the established obesity threshold [40]. Hyperglycemia was established in the MetS groups after STZ injection, as all rats had a FBG > 150 mg/dL [39].

*L. plantarum* Y_10b_ treatment resulted in a significant glucose-lowering activity in the MetS-treated rats; the FBG of the *L. plantarum* Y_10b_-treated MetS group was significantly reduced compared to the beginning of treatment and to the untreated MetS group, at the 2nd, 3rd, and 4th weeks of treatment. The FBG of the *L. plantarum* Y_10b_-treated MetS group was reduced to a level comparable to the control group on ND after 4 weeks of treatment.

The administration of *L. plantarum* Y_10b_ also significantly reduced obesity in the MetS group by the 3rd week of treatment, as indicated by a significant decrease in the Lee index compared with the untreated MetS control group. A similar reduction in the Lee index was recorded in a previous study after 8 weeks of treatment with LAB [96]. Also, the administration of *L. plantarum* Y_10b_ for 4 weeks significantly reduced the level of total cholesterol in the MetS group compared to the untreated MetS group (*p* < 0.0001); the cholesterol level of the probiotic-treated MetS group was comparable to that of the ND control group at the end of the treatment period.

Several studies have confirmed the positive effects of *Lactobacillus* species in lowering blood glucose, cholesterol levels, or both, in vivo [97, 98]. In the present study, *L. plantarum* Y_10b_ markedly improved MetS manifestations consistent with recent findings showing that *L. plantarum* strains alleviate obesity, insulin resistance, and dyslipidemia in high-fat-diet and MetS models [99]. Compared with those reports, *L. plantarum* Y_10b_ exhibited comparable or slightly stronger hypolipidemic and hypoglycemic activity, suggesting a strain-specific potency possibly related to its strong bile-salt-hydrolase activity and gut colonization ability. Human clinical studies and meta-analyses have likewise confirmed improvements in LDL-cholesterol and glycemic control following *L. plantarum* supplementation, although the magnitude of response varies depending on strain and treatment duration [100].

*L. plantarum* is among the qualified presumption of safety (QPS) list for microorganisms intentionally added to food or feed, supported by the European Food Safety Authority (EFSA) [101]. Further investigations are necessary to establish safety, including the absence of transmissible resistance determinants to clinically used drugs, the lack of harmful metabolic activity, such as D-lactate production, and the assessment of short- and long-term side effects in humans [102].

Several probiotic supplements containing *L. plantarum* are available in the market to support natural immunity and digestive health. Further research to elucidate the specific mechanisms underlying *L. plantarum* Y_10b_’s hypocholesterolemic and hypoglycemic effects should be performed, as well as complementary liver function tests to better elucidate the hepatic and metabolic impact of *L. plantarum* Y_10b_. Clinical trials to confirm the efficacy of *L. plantarum* Y_10b_ compared to placebo are necessary and could provide valuable insights for optimizing its use in both preventive and therapeutic contexts.

The study confirmed that Egyptian cuisine is a valuable, rich source of probiotic strains with diverse health-promoting and other beneficial characteristics, such as EPS production, hypocholesterolemic, hypoglycemic, anti-obesity, and antimicrobial activities. Therefore, high-throughput screening of probiotics from Egyptian foods and juices with evaluation of their in vivo activity and industrial applicability is highly encouraged in future studies.

## Conclusion

*L. plantarum* Y_10b_, a probiotic strain isolated from the Egyptian cottage cheese, is promising in alleviating the manifestations of MetS, with significant hypoglycemic, anti-obesity, and hypocholesterolemic effects in vitro and in vivo.

## Supplementary Information

Below is the link to the electronic supplementary material.

Supplementary File 1 (PDF 269 KB)

## Data Availability

All data generated and analyzed in the current study are available in the study and its supplementary materials.
