# Supplementary material for: A Potential Probiotic Lactiplantibacillus Plantarum Isolate from Egyptian Cottage Cheese Alleviates Metabolic Syndrome Manifestations: In Vitro and In Vivo Characterization
Source: Probiotics Antimicrob Proteins. 2026 Jan 24;18(5):6911–27. doi: 10.1007/s12602-025-10896-6 (PMC13368839; doi:10.1007/s12602-025-10896-6)
Supplement: Supplementary file 1 — (PDF 269 KB) [file 12602_2025_10896_MOESM1_ESM.pdf]

A probiotic *Lactiplantibacillus plantarum* isolate from Egyptian cottage cheese alleviates metabolic syndrome manifestations: in vitro and in vivo characterization

Journal: Probiotics and Antimicrobial Proteins

Yehya Abdel-Moniem[a,b], Kareem A. Ibrahim[b], Omneya M. Helmy[c], Mona T. Kashef[c,*]

[a] Post Graduate Program, Faculty of Pharmacy, Cairo University, Cairo 11562, Egypt

[b] Department of Microbiology and Immunology, Faculty of Pharmacy, Egyptian Russian University, Swiss road, Cairo 11829, Egypt

[c] Department of Microbiology and Immunology, Faculty of Pharmacy, Cairo University, Cairo 11562, Egypt

*** Corresponding author:** Mona T. Kashef

Email: mona.kashef@pharma.cu.edu.eg

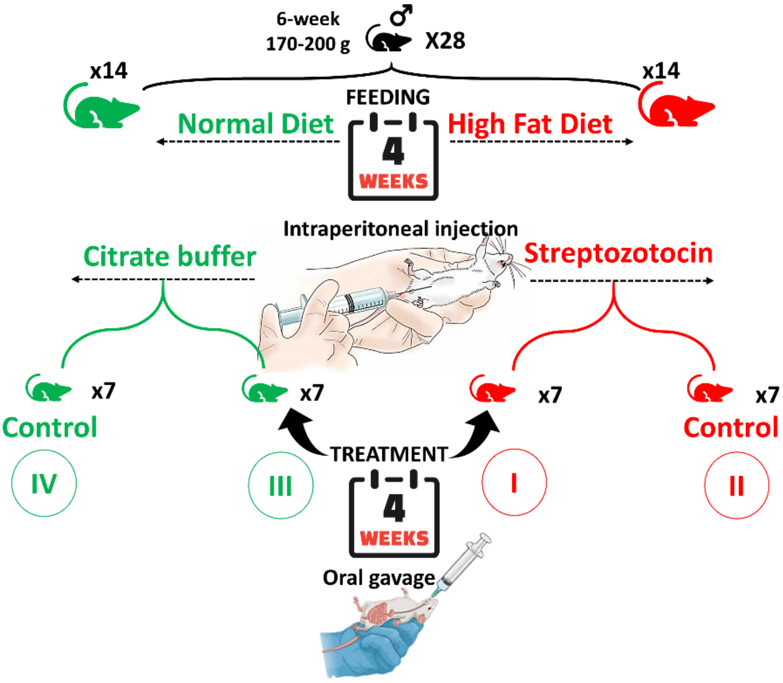

Supplementary Fig. 1 Experimental design showing the dietary and treatment interventions in the different male Wistar rats' groups
Male Wistar rats (n = 28) were divided into two main groups: Normal Diet (ND, n = 14) and High Fat Diet (HFD, n = 14), which were fed their respective diets for 4 weeks. Afterwards, each group was subdivided into two subgroups (n = 7 each): MetS groups (Groups I and II; HFD-fed) and Groups III and IV (ND-fed). The MetS groups were injected with STZ, while the ND-fed groups were injected with citrate buffer. Y_{10b} was administered to group I (HFD-fed MetS group) and group III (ND-fed control group), by oral gavage, for four weeks. The remaining two groups, group II (HFD-fed MetS) and group IV (ND-fed control), received normal saline as a control.
